# A Floristic Survey of Wild Edible Plants in Tuscan Maremma, Italy

**DOI:** 10.3390/plants14060976

**Published:** 2025-03-20

**Authors:** Mario Pentassuglia, Tiziana Lombardi, Giovanni Bambi, Irene Ventura, Benedetta D’Ambrosio, Andrea Bertacchi, Laura Pistelli

**Affiliations:** 1 Department of Agriculture, Food and Environment, University of Pisa, Via del Borghetto 80, 56124 Pisa, Italy; m.pentassuglia3@studenti.unipi.it (M.P.); g.bambi@studenti.unipi.it (G.B.); irene.ventura@phd.unipi.it (I.V.); b.dambrosio@studenti.unipi.it (B.D.); andrea.bertacchi@unipi.it (A.B.); laura.pistelli@unipi.it (L.P.); 2 Interdepartmental Research Centre “Nutraceuticals and Food for Health”, University of Pisa, Via del Borghetto 80, 56124 Pisa, Italy

**Keywords:** alimurgic flora, new foods, floristic studies, bioactive compounds, antioxidant activity, Tuscany

## Abstract

This study explores the floristic diversity of wild edible plants (WEPs) in the area surrounding Tirli, a small village in the Tuscan Maremma, Italy. Field surveys identified 128 vascular plant taxa across 46 families and 106 genera, with Asteraceae (26 taxa), Rosaceae (10 taxa), and Lamiaceae (8 taxa) being the most represented. The dominant life-forms are scapose Hemicryptophytes, scapose Therophytes, and rosulate Hemicryptophytes, with Euro-Mediterranean, Subcosmopolitan, and Steno-Mediterranean distributions prevailing. Statistical analyses revealed significant associations between life-forms and edible plant parts: scapose and rosulate Hemicryptophytes were linked to leaf use, scapose Therophytes to root use, and Phanerophytes to fruit use. The Asteraceae family exhibited exceptional versatility, being associated with various edible parts. Notably, the endemic species *Centaurea nigrescens* Willd. subsp. *pinnatifida* (Fiori) Dostál was recorded for the first time in the Tuscan Maremma, underscoring the area’s naturalistic value. Traditional culinary practices were linked to some edible plants, which were analyzed for bioactive compounds, including photosynthetic pigments, primary metabolites, secondary metabolites, and antioxidant activity. The results confirmed their biochemical richness and functional properties. This study emphasizes the ecological, nutritional, and cultural significance of Tirli’s wild edible flora, promoting biodiversity conservation, cultural heritage preservation, and sustainable food practices.

## 1. Introduction

Wild edible plants (WEPs) are defined as uncultivated and undomesticated plants that grow in their natural environment and provide edible components such as roots, leaves, or fruits for human consumption [1]. WEPs have played a crucial role in human history, providing essential resources for survival and social development on a global scale [2]. WEPs continue to be consumed in many countries and territories of the world, not only in subsistence economies but also in rural and even urban areas of developed countries [3,4]. The number of species consumed globally, however, remains rather uncertain [2], and the overall understanding of their use is often fragmented and incomplete, which is also due to the gradual decline of traditional knowledge related to their use [5].

Modernization, consumerism, and disinterest among younger generations are leading to a significant loss of this cultural heritage [6]. This phenomenon represents a critical challenge, as WEPs not only contribute to dietary diversification but also serve as strategic resources for addressing global issues such as food security and biodiversity conservation. These topics are central to international discussions, as highlighted by the Convention on Biological Diversity [7,8]. Consequently, it is widely recognized that this heritage must be preserved, as it represents cultural, ecological, and scientific value [9]. The importance of WEPs extends beyond nutritional value; many wild edible species possess nutraceutical and bioactive properties that are vital for health and well-being [5,10,11]. Due to these properties, WEPs are increasingly recognized as novel functional foods, attracting growing attention and research efforts [11,12].

According to the International Food Information Council (IFIC), functional foods are defined as foods or dietary components that may provide a health benefit beyond basic nutrition [5]. Awareness of the nutraceutical potential of WEPs has been steadily increasing [13,14]. These plants are rich in secondary metabolites, which play a crucial role in preventing nutritional deficiencies and protecting against chronic diseases [15]. The bioactive compounds found in WEPs represent promising candidates for the development of nutraceuticals, with significant potential to improve human health [16,17].

Phytoalimurgy is a branch of botany that focuses on the rediscovery and study of wild plants for their nutritional value [18,19], and it emerged in response to urgent food needs. This discipline was formalized in the 18th century with Giovanni Targioni-Tozzetti’s treatise “*De alimenti urgentia*”, and it has now taken on a central role in promoting food diversification and greater awareness of natural resources and human nutrition [20,21]. Studies aimed at the census of WEPs at the local level, together with their conservation and sustainable management, thus become extremely important and serve as fundamental starting points for ensuring future food security and maintaining ecological balance [1].

Italy, with its extraordinary floristic diversity, provides an ideal context for studying wild edible plants (WEPs) in the Mediterranean region. In this context, the impacts of climate change—such as soil salinization—are increasingly leading to the recognition of many halophytic species as valuable food resources [22,23]. The Italian vascular flora include 7591 taxa [24], of which 14.53% are represented by known edible species [25]. Located in Tuscany, the second-richest region in flora after Piedmont [24], the Maremma is one of the most ecologically and scenically relevant areas in Italy. The Maremma is an area of extraordinary environmental value, characterized by diverse ecosystems including wetlands, coastal halophytic communities, holm oak forests, Mediterranean scrub, and garrigue [26]. Due to its geomorphological characteristics, this territory has historically seen limited human activities, resulting it being less developed and less populated compared to other regions of Italy. This has contributed to the preservation of natural ecosystems and the development of significant biodiversity [27]. Despite the extensive literature available for Tuscany’s edible flora, no scientific publications provide a detailed overview of the WEPs present in the Maremma [19,25,28,29]. However, these plants are not uncommon in the region’s culinary tradition. One notable example is the traditional Maremma soup *Acquacotta*, which, depending on the season, may include species such as *Beta vulgaris* L. subsp. *maritima* (L.) Arcang., *Clinopodium nepeta* (L.) Kuntze subsp. *nepeta*, *Cichorium intybus* L., *Urtica dioica* L., *Sonchus* sp.pl., and *Portulaca oleracea* L. [30].

This work is part of the activities outlined in the B.E.T.A project (Measure 19.2 Specific Leader Action “Community Regeneration Projects”), funded by the Tuscany Region, and it represents a preliminary contribution to the knowledge of wild edible plants (WEPs) in this area of Tuscany, with particular attention to the territory surrounding the small village of Tirli, located in northern Maremma. Thanks to its geographical position and historical context, Tirli has preserved a rich floristic heritage, making it an ideal context for in-depth study of the interaction between humans and wild edible plants.

Through the census of WEPs and biochemical analyses conducted on species selected based on some community interviews, this work not only documents local biodiversity, but also contributes to the enhancement of natural resources and the rediscovery and preservation of ancient knowledge. Its aim is to fill a gap in the scientific literature and provide insights for future research on the phytoalimurgical heritage of the area.

## 2. Results

### 2.1. Floristic Inventory

The edible wild flora identified through field surveys in the studied area amount to 128 taxa (species and subspecies). These taxa are reported in a floristic list, where each species is categorized within its family and accompanied by its biological form following the Raunkiaer system [31], chorotype, and edible plant parts (Supplementary Table S1). They are distributed across 46 families and 106 genera.

**Figure 1:**
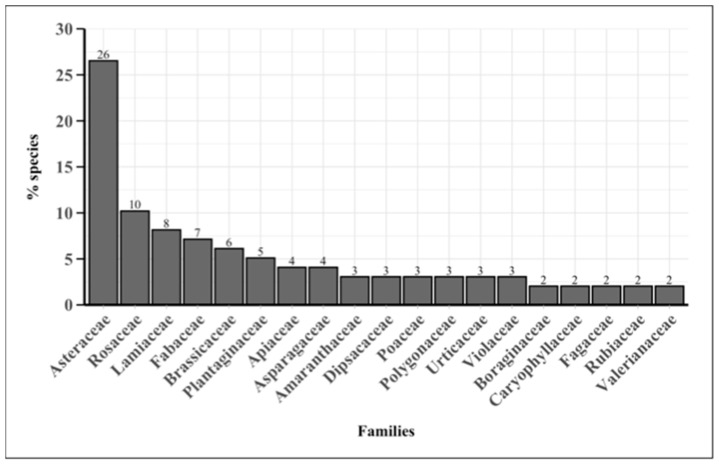
Bar plot showing the percentage of most representative families across the inventory of WEPs (frequency > 2). Numbers above the bars indicate the number of species.

The most represented families, with more than three species, are Asteraceae (26 taxa), Rosaceae (10 taxa), Lamiaceae (8 taxa), Fabaceae (7 taxa), Brassicaceae (6 taxa), Plantaginaceae (5 taxa), Apiaceae (4 taxa), and Asparagaceae (4 taxa) (Figure 1). The most representative genera in terms of the number of species are *Crepis* (3), *Plantago* (3), *Rumex* (3), *Sonchus* (3), and *Viola* (3).

### 2.2. Life-Form Spectrum

**Figure 2:**
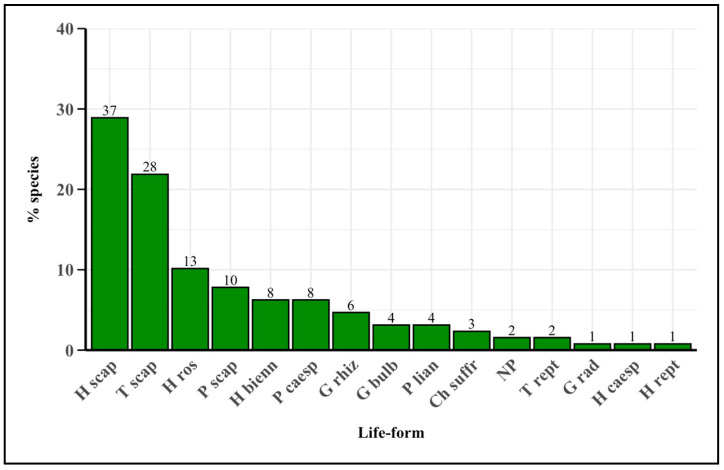
Bar plot graph of life-forms and their subdivisions of all the edible flora. Numbers above the bars indicate the number of species. H scap = scapose Hemicryptophytes; T scap = scapose Therophytes; H ros = rosulate Hemicryptophytes; P scap = scapose Phanerophytes; H bienn = biennial Hemicryptophyte; P caesp = caespitose Phanerophytes; G rhiz = rhizomatose Geophytes; G bulb = bulbous Geophytes; P lian = lianas Phanerophytes; Ch suffr = suffruticose Chamaephytes; NP = Nano-Phanerophytes; T rept = reptant Therophytes; G rad = radiciform Geophytes, H caesp = caespitose Hemicryptophyte; H rept = reptant Hemicryptophyte.

The life-form spectrum (Figure 2) reveals that the most represented species are scapose Hemicryptophytes, with a total of 37 species. These are followed by scapose Therophytes, with 28 species, and rosulate Hemicryptophytes, with 13 species. Other significant life-forms include scapose Phanerophytes, biennial Hemicryptophytes, and caespitose Phanerophytes. Rhizomatous Geophytes and bulbous Geophytes are less represented. Lianous Phanerophytes and other life-forms each account for fewer than four species.

### 2.3. Chorological Spectrum

**Figure 3:**
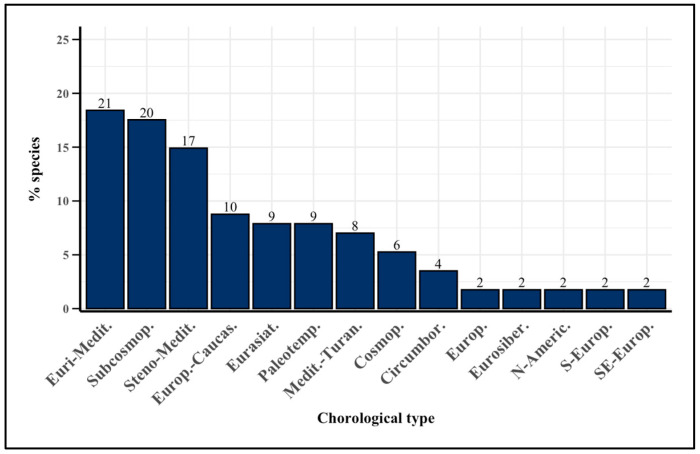
Bar plot of the most representative chorological spectra across the inventory of WEPs (frequency > 2). Numbers above the bars indicate the number of species. Euri-Medit. = Euromediterranean; Subcosmop. = Subcosmopolitan; Steno-Medit. = Stenomediterranean; Europ-Caucas. = Europe-Caucasian; Paleotemp. = Paleotemperate; Medit-Turan. = Mediterranean-Turanian; Cosmop. = Cosmopolitam; Circumbor. = Circumboreal; Europ. = European; Eurosiber. = Eurosiberian; N-Americ. = North American; S-Europ. = Southern European; SE-Europ. = Southeastern European.

The chorological spectrum (Figure 3) reveals a predominance of Euro-Mediterranean distributions (21 species) and Subcosmopolitan distributions (20 species), followed by Steno-Mediterranean distributions (17 species). Other significant chorological types include Euro-Caucasian (10 species), Eurasian (9 species), Paleotemperate (9 species) and Mediterranean-Turanian (8 species). Cosmopolitan (6 species) and Circumboreal (4 species) distributions were less represented.

The smallest common types included European, Eurosiberian, North American, South European, and Southeast European distributions, each with two species.

### 2.4. Ellenberg Indicator Values

**Figure 4:**
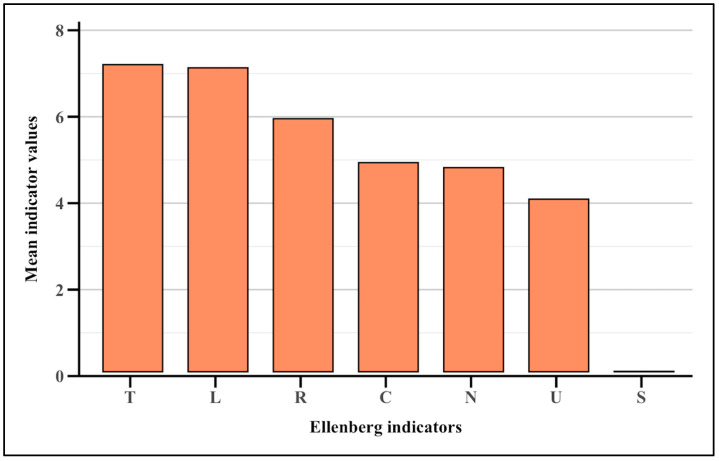
Bar plot graph of the Ellenberg ecological indicators (mean values) for all the edible flora. T = Temperature; L = Light; R = Soil Reaction; C = Continentality; N = Soil Nutrients; U = Soil Moisture; S = Salinity.

The mean Ellenberg indicator values for all the wild edible flora (Figure 4, Supplementary Table S2) are temperature (7), light (7), soil reaction (6), continentality (5), nitrogen (5), moisture (4), and salinity (0).

### 2.5. Biochemical Analyses

Table S3 reports the data on the biochemical compounds of selected WEPs. The values for total chlorophyll (the sum of chlorophyll *a* and chlorophyll *b*) showed a significative difference between species, which ranged from 1.73 mg/g DW in *Myrtus communis* L. and 1.99 mg/g DW in *Plantago coronopus* L. to 20.23 mg/g DW in *Sonchus oleraceus* L. and 15.65 mg/g DW in *Cichorium intybus* L. Other species with high chlorophyll content were *Umbilicus rupestris* (Salisb.) Dandy (15.62 mg/g DW) and *Campanula rapunculus* L. (15.51 mg/g DW). The ratio of chlorophyll *a* to chlorophyll *b* (CHL*a*/CHL*b*) was also determined. Most leaves showed the ratio between 1.75 and 2.84, which indicated the habitual status of wild-grown plants, although few of them exhibited a significantly lower ratio (0.87 for *Clinopodium vulgare* L. subsp. *vulgare*, 0.93 for *Myrtus communis*, and 1.31 for *Laurus nobilis* L.)*,* probably due to the environmental conditions and the period of harvest [32], as demonstrated by the observed significant variation in moisture content. In particular, 20 out of the 28 analyzed species exhibited notably high moisture content (over 80%) typical of herbaceous plants, whereas the lowest values were observed in the shrubs *Myrtus communis*, with 59.22%, and *Laurus nobilis*, with 57.1%.

To complete the analyses of pigments, the total content of carotenoids (also including xanthophylls) was determined; the results exhibited significant variability among the species, ranging from 0.13 to 2.67 mg/g DW. The species with the higher carotenoid concentration was *Sonchus oleraceus*(2.45 mg/g DW). Conversely, the species with the lowest or negligible carotenoid content included *Myrtus communis* (0,13 mg/g DW), *Clinopodium vulgare* subsp. *vulgare* (0.28 mg/g DW), *Plantago coronopus* (0.31 mg/g DW), *Laurus nobilis* (0.47 mg/g DW), and *Borago officinalis* L. (0.61 mg/g DW).

The primary metabolites, such as total soluble reducing sugars (mainly represented by glucose and fructose), ranged from a minimum of 31.03 mg GLU/g DW in *Beta vulgaris* L. subsp. *maritima* (L.) Arcang. to a maximum of 130.47 mg GLU/g DW in *Laurus nobilis*. High sugar content was also found in species including *Hypericum perforatum* L. subsp. *veronense* (Schrank) Ces. (97.82 mg GLU/g DW) and *Myrtus communis* (81.49 mg GLU/g DW). Conversely, species such as *Allium triquetrum* L. and *Urtica dioica* L. exhibited significantly lower levels, with 33.94 and 35.45 mg GLU/g DW, respectively.

The protein content expressed as a percentage indicated *Clematis vitalba* L., *Plantago coronopus*, and *Myrtus communis* as the species with a very low content (4.59, 6.90 and 7.5%, respectively), while *Malva sylvestris* and *Sambucus nigra* L. were found to have the highest percentage (36.14 and 31.29%, respectively).

The levels of polyphenols and flavonoids were determined for their role as antioxidant compounds. The total polyphenol content was higher than 100 mg GAE/g DW in *Myrtus communis* (146.81 mg GAE/g DW) followed by *Clinopodium vulgare* subsp. *vulgare* (137.14 mg), *Hypericum perforatum* subsp. *veronense* (111.23 mg GAE/g DW), *Poterium sanguisorba* L. subsp. *sanguisorba* (128.63 mg GAE/g DW), and *Plantago coronopus * (109.83). Other species, such as *Chenopodium album* L. subsp. *album*, *Allium triquetrum*, and *Malva sylvestris* L., showed comparatively lower concentrations in the range 16.49–20.13 mg GAE/g DW. The distribution of polyphenols highlights the diversity among plant families, with the Lamiaceae and Asteraceae families exhibiting particularly notable values. A similar behavior between the species was observed for the flavonoid concentration (a subclass of polyphenols) with the highest value in *Clinopodium vulgare* subsp. *vulgare* and the lowest content in *Allium triquetrum* (134.16 mg CE/g DW and 4.78 mg CE/g DW, respectively).

Antioxidant activity represents the major property of the class polyphenols and carotenoids. The activity was measured by two methods, the ABTS (antioxidant capacity) assay and DPPH scavenging activity, both determined as the mM concentration of Trolox equivalent (TE) per gram of dry weight (DW). The ABTS results showed considerable variability in antioxidant capacity: a very low activity was observed in *Allium triquetrum*, *Chenopodium album* subsp. *album* and *Malva sylvestris* L. (around 3 mM TE/g DW) while higher activity (13.71 mM TE/g DW) was found in *Reichardia picroides* (L.) Roth., followed by *Laurus nobilis, Borago officinalis*, and *Crepis leontodontoides* All. (12.70, 2.32, and 12.01 mM TE/d DW, respectively). The DPPH antioxidant capacity assay confirmed the low activity in *Allium triquetrum* (1.17 mM TE/g DW) and the high activity in *Laurus nobilis* 11.89 mM TE/g DW). Other interesting results highlighted the relatively lower antioxidant activity in *Beta vulgaris* subsp. *maritima* and the high antioxidant activity in *Hypericum perforatum* subsp. *veronense* and *Clinopodium nepeta* (L.) Kuntze subsp. *nepeta*.

### 2.6. Statistical Analyses

#### 2.6.1. Heatmap Visualization: Insights from MCA Method into Life-Form and Chorological Type Interactions

**Figure 5:**
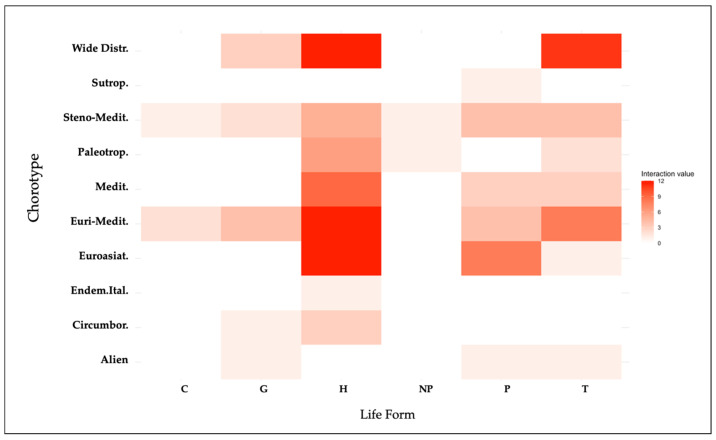
Heatmap visualization of the biogeographical distribution and life-form with their interaction value. The color gradient effectively illustrates the varying degrees of interaction values, with red indicating stronger relationships and white indicating weaker or no significant interactions.

The heatmap visualization (Figure 5) reveals that Hemicryptophytes are predominantly associated with a Mediterranean chorotype, alongside significant links to Eurasian and wide distributions (Cosmopolitan and Subcosmopolitan). Therophytes, on the other hand, are primarily linked to a wide distribution. Phanerophytes show a stronger association with Eurasian distribution. The remaining life-forms, which are associated with a smaller number of species within the analyzed WEPs, demonstrate more uniform geographical distributions in comparison to the more predominant one. This pattern underscores the diverse biogeographical preferences of various life-forms categories and their specific geographical associations.

#### 2.6.2. Multiple Correspondence Analysis (MCA) to Identify Interaction Patterns

**Figure 6:**
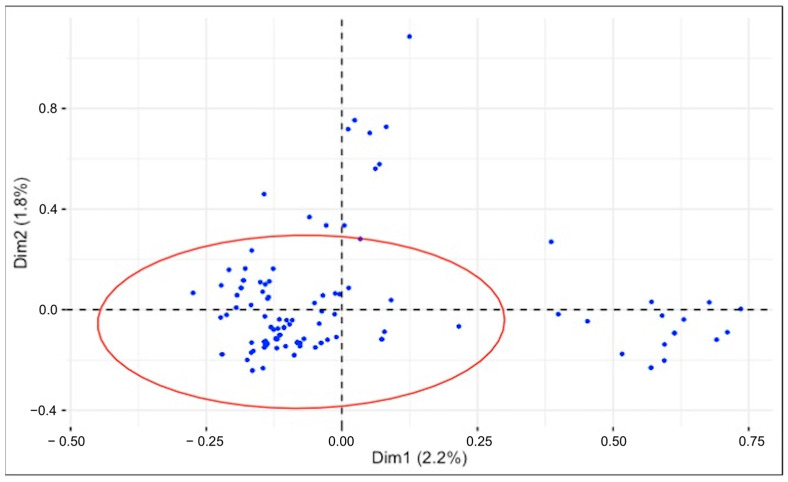
Species distribution with 95% confidence ellipse in MCA.

Multiple correspondence analysis (MCA) shows the distribution of the species on the first two main dimensions, with a 95% confidence ellipse indicating the area where most observations are concentrated (Figure 6). The ellipse represents the central area of the observations, illustrating some cohesion between species and suggesting the sharing of similar characteristics according to the categorical variables analyzed. The first two dimensions account for only 2.2% and 1.8% of the total variance, respectively. These low percentages reflect the complexity and dispersion of the dataset but may also be attributed to the nature of MCA of binary datasets, where the total inertia is often artificially inflated, causing the explained variance of the first dimensions to be underestimated [33]. Despite these low explanatory percentages, the plot provides an initial framework for visualizing the relationships and grouping patterns. Further dimensions and complementary analyses were explored to better understand the structure of the data.

**Figure 7:**
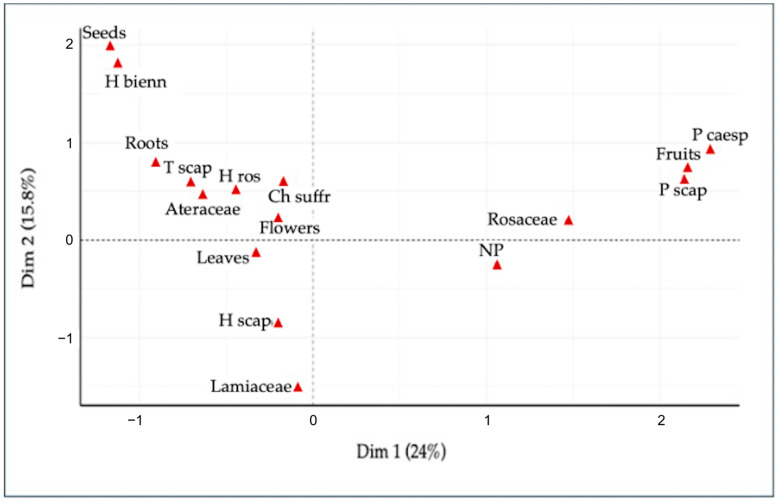
Selected Key Variables and Their Associations.

For a clearer visualization, only the most influential variables contributing to the clustering and separation of species were highlighted (Figure 7). The selected variables exhibit the highest contribution to the first two principal dimensions (Dim1 and Dim2), which in this case account for 24% and 15.8% of the total variance, respectively. The resulting plot illustrates the distribution of variables within the multidimensional space.

#### 2.6.3. Principal Component Analysis (PCA) and Hierarchical Cluster Analysis (HCA) of Phytonutritional Compounds

**Figure 8:**
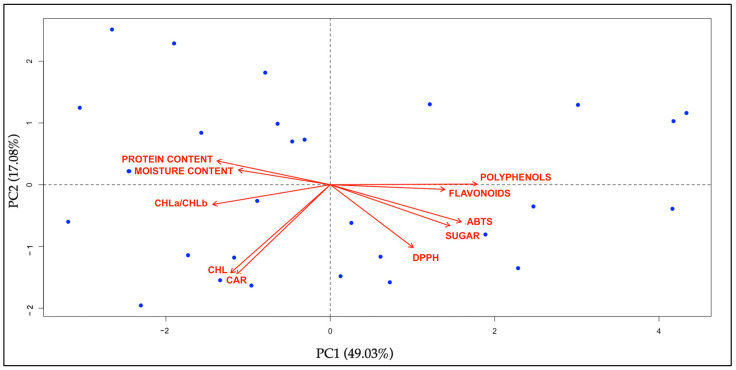
Principal component analysis (PCA) was performed on phytonutritional compounds (both primary and secondary metabolites) and antioxidant activities of the twenty-eight edible leaves.

PCA and HCA analyses were performed on both primary and secondary metabolites, as well as on the antioxidant activity of twenty-eight plants commonly used by the local community. In the PCA (Figure 8), the species are distributed across the four quadrants, indicating that the aggregation varies among the variables. This significant separation is due to the differing contents of compounds in the plant material. The results highlight a clear distinction between moisture and protein content in the upper left quadrant while in the lower quadrants are shown the secondary metabolites with antioxidant activity, as well as sugar content separated from the photosynthetic pigments.

**Figure 9:**
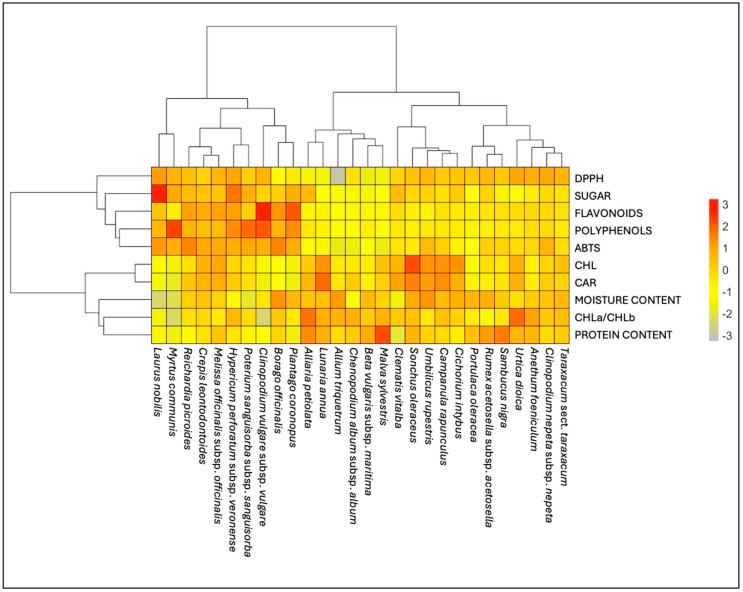
Hierarchical cluster analysis (HCA) of phytonutritional compounds (both primary and secondary metabolites) and antioxidant activities of the twenty-eight edible leaves. DPPH = DPPH assay; ABTS = ABTS assay; CHL = total chlorophyll; CAR = total carotenoids.

This behavior strengths the results in the dendrogram of the HCA (Figure 9) showing two main clusters: one includes the moisture content, protein percentage, and photosynthetic pigments, while the second one includes the soluble sugar content, the secondary metabolites, and their antioxidant activity. However, analyzing the species presents a great challenge. The left part of the HCA reveals ten species that have the highest amounts of most metabolites and exhibit the highest antioxidant activity.

## 3. Discussion

### 3.1. Floristic Inventory

The WEPs of Tirli include a remarkable biodiversity of plant families (Figure 1), reflecting the ecological and geomorphological traits of the study area [34,35]. The predominance of families such as Asteraceae (26.5%), Rosaceae (10.2%), and Lamiaceae (8.2%) aligns with what has already been observed regarding the distribution of most edible plants in the Mediterranean region. These families, well adapted to local climatic conditions, are distinguished by the diversity of their edible plant parts [36,37,38,39]. This prevalence may also reflect the relative floristic dominance, along with a possible cultural heritage, supported by the distinctive botanical and phytochemical characteristics of plants that are easily recognizable for their showy flowers, intense aromas, and unique flavors [40]. Numerous studies have confirmed that Asteraceae are among the most widely used plants in Italy [29,30,41,42], especially in the island regions of the country [43]. The high presence of edible Asteraceae appears to be linked to the large number of species within the Italian flora [24] in addition to their availability across multiple seasons, with the basal rosette remaining accessible even in autumn and winter [25]. The notable presence of some genera, such as *Crepis* spp., *Plantago* spp., *Rumex* spp., *Sonchus* spp., and *Viola* spp., belonging to the Asteraceae, Plantaginaceae, Polygonaceae, and Violaceae families, also suggests an affinity for habitats characterized by open spaces, such as grasslands and ruderal areas, which are typical of the Mediterranean region [25]. These genera are not only ecologically versatile but also nutritionally significant, contributing to the dietary diversity and providing multiple health benefits to local communities [44,45,46,47]. The use of *Sonchus* species, for example, is supported by their high content of vitamin C, carotenoids, and omega-3 fatty acids, making them valuable for their antioxidant and anti-inflammatory properties [48]. Similarly, *Rumex* spp. is recognized for its rich composition of beneficial metabolites, including polyphenols, flavonoids, and organic acids, which contribute to its traditional use for both nutritional and medicinal purposes [49]. Their widespread use underscores the interplay between ecological availability and cultural preferences, as well as the adaptability of these taxa in diverse environmental conditions. Overall, this floristic composition provides valuable insights into the local biodiversity and the potential food uses of the plant species present.

### 3.2. Life-Form Spectrum

The life-form spectrum highlights a significant predominance of scapose Hemicryptophytes (28.9%) (Figure 2). This life-form is characterized by having the highest number of species in European vegetation, a success attributed to its remarkable taxonomic and functional diversity [50]. According to Raunkiær’s system [31], they are herbaceous perennial plants with buds located at the soil surface. Hemicryptophytes form a highly diverse group and are the most widespread life-form in the flora of humid temperate climates [51]. The availability of the rosette foliage of certain species, according to the relevant presence of rosulate Hemicryptophytes (10.2%), makes them particularly suitable for culinary use. Therophytes, which consist mostly of scapose plants (21.9%), are primarily valued for their tender leaves and ease of harvesting, making them frequently consumed. In some cases, their aerial parts may also be utilized. Therophytes are defined as species with a limited vegetative cycle that thrive in soils that are well drained and low in nutrients. They survive in the adverse season in the seed stage and complete their life cycle in the favorable season [52,53]. Phanerophytes, including woody perennial plants with buds located above the ground surface, are represented by scapose plants (7.8%) such as *Myrtus communis* L., *Ficus carica* L., *Prunus avium* (L.) L., *Pyrus communis* L., and *Olea europaea* L. They have the highest proportions in Mediterranean scrub and a biodiversity associated with the presence of forests and wetlands. This life-form is among the most widely used, primarily because their fruits and seeds are extensively consumed in both traditional and popular culinary practices [50].

### 3.3. Chorological Spectrum

The chorological spectrum underscores the prevalence of Euri-Mediterranean, Steno-Mediterranean, and Subcosmopolitan species, thereby confirming the patterns identified in the biological spectrum and aligning with the climatic conditions of the study area (Figure 3). Eurasian species are also prominently represented. Noteworthy is the presence of an endemic species identified in accordance with Pignatti [54] as *Centaurea nigrescens* Willd. subsp. *pinnatifida* (Fiori) Dostál. In this study, we report the first record in the Maremma territory, specifically regarding its presence in the study area. This finding enriches the floristic knowledge of the region and provides new insights for future investigations into its ecological potential and adaptation to local environmental conditions.

From a phytogeographical perspective, it is interesting to note that most of the food flora originate from the Mediterranean region, with a significant presence of Euri-Mediterranean (18.4%) and Steno-Mediterranean plants (14.9%). This highlights the deep connection between the diverse cultures of the Mediterranean area. In fact, several plant species are used across different regions of Italy and throughout the Mediterranean, emphasizing the cultural and culinary exchanges that have shaped this rich botanical and culinary heritage [43]. The presence of Subcosmopolitan (17.5%) species in the local food flora may be attributed to their broad ecological tolerance and ability to adapt to a wide range of environments, a trait that has made them an integral part of various culinary traditions in the Mediterranean region. Among the WEPs characterized by a widespread distribution, Subcosmopolitan species are the most representative in Italy [25], confirming a major use of agricultural and urban land with respect to the natural environments [55]. The high incidence of Eurasian species is due to the strong bio-geographical connections between the flora contingent in the Italian Apennine region and areas belonging to Eurasia, indicating that the study area has a wetter microclimate with cold, wet winters and hot but not dry summers [56].

### 3.4. Ellenberg Indicator Values

The mean Ellenberg indicator values [57] provide valuable insights into the ecological preferences of the plant species and environmental conditions of the study area. The results reveal a floristic composition adapted to warm, temperate climates, with well-balanced ecological conditions (Figure 4, Supplementary Material Table S2).

The temperature indicator (T = 7) highlights vegetation adapted to warm and temperate climates, typical of Mediterranean mountainous regions. In accordance with the chorological spectrum, Euri-Mediterranean species are prevalent. Similarly, the light indicator (L = 7) suggests a predominance of species thriving in well-lit environments, such as grasslands or forest edges, with a minor presence of species tolerant of reduced light.

The soil reaction indicator (R) shows intermediate values, indicating mesophilic species that prefer neutral to slightly basic soils, excluding strongly acidic or very basic environments. This aligns with moderately calcareous soils, often found in hilly or plain areas.

The continentality indicator (C) further reflects a temperate climate, characteristic of regions in transition between the Mediterranean and continental zones, showcasing the flexibility of the flora in responding to climatic gradients.

The high soil nutrient value (N) suggests that species predominantly grow in fertile, well-humified soils, typical of agricultural areas or moderately enriched habitats. This underscores the soil’s capacity to support robust plant productivity.

Similarly, the intermediate soil moisture value (U) indicates species thriving in moderately dry but adequately hydrated soils, avoiding extremes such as flooding or excessive aridity, thus reflecting a stable water balance.

Finally, the salinity indicator (S) confirms the absence of species tolerant to salinity, consistent with the ecological characteristics of the area, which do not include saline habitats. However, *Beta vulgaris* L. subsp. *maritima* (L.) Arcang. is the only species tolerant of low salt concentrations. The presence of this species in a hilly context like Tirli highlights its remarkable ecological adaptability. This makes it particularly interesting not only from a botanical perspective but also for its potential role in transitional ecosystems or moderately stressed soils. Its importance thus extends to both biodiversity conservation and the enhancement of edible wild plant resources, aligning with the goal of sustainable land management.

### 3.5. Exploring Life-Form and Chorological Type Interactions Through Heatmap Visualization

The heatmap clearly visualizes the typical biogeographical distributions of different life-forms (Figure 5). Hemicryptophytes tend to be associated with chorological types that reflect an adaptation to variable and less stable climatic conditions, such as the Euri-Mediterranean and wide distribution groups. Therophytes, on the other hand, show a stronger association with wide distribution, indicating their ability to adapt to more diverse climatic and geographical conditions. Phanerophytes, finally, are closely linked to Eurasian distribution, suggesting an adaptation to more stable climates with less seasonal variability. This visualization is crucial for selecting wild edible plants best adapted to local ecological and geographical conditions, providing preliminary but useful results, particularly in rural contexts characterized by high climatic variability [58].

### 3.6. Multiple Correspondence Analysis (MCA): From Species Distribution to Key Variable Associations

The multiple correspondence analysis highlighted significant associations between plant families, life-form, and plant edible parts, particularly the roots, leaves, fruits, seeds, and flowers (Figure 6). The species association plot shows the distribution of observations (species) in the space defined by the two main dimensions (Dim1 and Dim2). The distribution is centered around the origin, with some observations diverging, indicating atypical combinations of variables. The ellipse is the central area of the observations, where combinations of variables tend to be more frequent. Observed outliers are likely to be associated with extreme categories.

Based on the variance explained by the first two dimensions of the MCA, we selected the variables with the highest contributions, visualizing a clearer interpretation of the data structure. In the key variables plot, the most significant contributors to the first dimension include fruits, scapose Phanerophytes (P scap), and cespitose Phanerophytes (P caesp), all of which are in the same quadrant. Phanerophytes are closely associated with fruit production, suggesting that this combination characterizes certain analyzed species. Similarly, other biological forms, such as biennial Hemicryptophytes (H bienn), show a relationship with the use of seeds, positioning themselves in contiguous areas within the factorial plane.

A clear association between scapose Therophytes (T scap) and the food use of roots emerges. This relationship may reflect an ecological adaptation of Therophytes, annual plants with a short life cycle, whereby they accumulate important resources in their roots to survive in challenging or highly seasonal environments.

Likewise, a significant relationship between Hemicryptophytes, specifically rosulate Hemicryptophytes (H ros) and scapose Hemicryptophytes (H scap), and the use of leaves can be observed. This connection is consistent with the ecological and morphological response of Hemicryptophytes, which often exhibit basal rosettes or scapose structures that facilitate the availability and collection of leaves. Leaves, in these life-forms, not only play a crucial role in photosynthetic metabolism but also serve as a traditional food resource in regions where these plants are present.

The association between suffruticose Chamaephytes and flowers is particularly notable, as it underscores a specific ecological role.

Chamaephytes show high biodiversity in the Mediterranean as well as in the boreal region. They are typically characterized by the ability to survive low temperatures and adapt to specific habitats [31,50]. They often have flowers, which are significant for pollination. The most widely used parts of Chamaephytes in Italy, especially aromatic suffruticose plants, are the flowers and leaves [25].

The placement of Nano-Phanerophytes (NPs), such as *Lavandula stoechas* L. subsp. *stoechas* and *Rosa canina* L., reflects their unique morphological and ecological characteristics, which influence their distribution in relation to other variables. Their location away from the origin of other categories suggests that these plants are clearly distinct due to their unique adaptive strategies.

Plant families reveal specific associations with biological forms and utilized plant parts (Figure 7). For example, Rosaceae occupies a distinct position along the second dimension, indicating a unique relationship with traits such as fruit utilization. The value of the edible component of this family stems from the crucial role its fruits play in food-related activities and its economic significance [59]. In contrast, Asteraceae is associated with the use of leaves, flowers, and roots. Being close to the origin, this family presents a more generalized distribution, without a strong association with specific categories along the first two dimensions. Several ethnobotanical studies [36,60,61] highlight that the Asteraceae family is among the most represented in wild alimurgical flora. This prominence is attributed to their remarkable ecological adaptability and the diversity of the plant parts utilized, including the leaves, roots, and flowers. The proximity to the category of the scapose Hemicryptophytes suggests an association with a specific life-form.

The absence of correlations with plant parts such as fruits and seeds indicates that species belonging to the Lamiaceae family are mainly used for other parts, such as leaves or flowers, which are often used for culinary and medicinal purposes. This reflects their role in the ecosystem and their use in traditional practices.

In conclusion, the first dimension (Dim1) mainly separates categories related to edible plant parts (fruits, seeds, leaves, flowers) and associated life-forms (e.g., H bienn, P scap).

The second dimension (Dim2) seems to further highlight the relationship between specific life-forms and plant families, such as Asteraceae, Lamiaceae, and Rosaceae. Families like Rosaceae show a clear association with fruit use, while Asteraceae and Lamiaceae are mainly linked to leaf and flowers, which are characterized by a more common and wider distribution. These findings provide a foundation for a better understanding of ecological and edible part relationships, contributing to knowledge about the WEPs used by humans.

### 3.7. Biochemical Analyses

The results of this study provide a comprehensive overview of the primary and secondary metabolites in 28 species from 20 botanical families, highlighting significant variability in total chlorophyll, carotenoids, sugars, and other bioactive compounds (Figure 8 and Figure 9, Supplementary Table S3). These findings expand on the existing literature and underscore the nutritional and functional potential of these species. Among the species analyzed, *Sonchus oleraceus* L. exhibited the highest total chlorophyll content and a notable level of carotenoids. These results are consistent with prior studies identifying *S. oleraceus* as a rich source of bioactive compounds, including carotenoids and polyphenols, with antioxidant and anti-inflammatory properties [62]. Furthermore, its high vitamin C, vitamin A, and fiber content reinforces its value for dietary and functional applications [63]. Other species, such as *Cichorium intybus* L. and *Campanula rapunculus* L., also demonstrated significant chlorophyll content, confirming their nutritional relevance.

These findings are aligned with previous observations that many Asteraceae species tend to have elevated chlorophyll and carotenoid levels [64]. An important physiological trait is the ratio of chlorophyll *a* to chlorophyll *b* (CHL*a*/CHL*b*), which is used as an indicator of photosynthetic adaptations. This trend is consistent with other Asteraceae species, which frequently exhibit elevated CHL*a*/CHL*b* ratios [65]. A recent study [32] reported that the ratio of CHL*a*/CHL*b* and the ratios of chlorophyll/carotenoids and xanthophylls are related to the difference between C3 and C4 plants but are also strictly linked to the environmental conditions of the plants, including solar radiation, water availability, soil condition (physical, chemical, and biological characteristics), and, finally, the stage at harvest. In general, this work was primarily conducted during the flowering stage in the spring and summer seasons, which in the Mediterranean area correspond to high temperatures and limited water availability. It is noted that there is a direct correlation in the species *Laurus nobilis* L. and *Myrtus communis* L. between the moisture content and some compounds, such as chlorophylls, carotenoids, and sugars: a low percentage of water in the leaves is associated with a lower content of pigments, but a high sugar content can contribute to osmotic adjustment to counteract some water stress during some seasonal conditions [66]. The study identified *Laurus nobilis* as a species that has a high sugar content but also provides a good source of other bioactive compounds such as polyphenols and flavonoids, confirming what has already been reported by other researchers [67]. Our findings regarding another plant showing a high content of sugars, namely, *Hypericum perforatum* L. subsp. *veronense* (Schrank) Ces., are supported by previous studies suggesting that Mediterranean species, particularly within the Asteraceae family, are rich in simple and complex carbohydrates, enhancing their nutritional value [64].

Secondary metabolites such as polyphenolic compounds and the main class of flavonoids are recognized as important nutraceutical properties and have been determined in the leaves of alimurgic plants. Secondary metabolites, particularly polyphenols and flavonoids, play a critical role in determining the antioxidant potential of these species. *Clinopodium vulgare* L. subsp. *vulgare* is particularly rich in polyphenols, such as rosmarinic and ellagic acids, as well as in flavonoids, with the highest flavonoid content among the species analyzed. This highlighted the antioxidant properties of *Clinopodium vulgare* subsp*. vulgare* derived from its secondary metabolites [68].

The polyphenol content varied widely across the species and families such as Myrtaceae and Lamiaceae showed particularly high levels. *Myrtus communis* was noted for its strong antioxidant potential, corroborating findings linking high polyphenol content to enhanced radical-scavenging activity [69]. Conversely, *Chenopodium album* L. subsp. *album* exhibited low polyphenol levels, consistent with reports of variability across wild and cultivated species [70]. Antioxidant activity, assessed via ABTS and DPPH assays, varied significantly among species. The highest ABTS activity was observed in *Reichardia picroides* (L.) Roth while *Laurus nobilis* recorded the highest DPPH activity. These results are consistent with the general correlation between polyphenol and flavonoid content and antioxidant capacity. For instance, *Anethum foeniculum* L. demonstrated moderate polyphenol levels and antioxidant activity, consistent with its phenolic and flavonoid profile [71]. Similarly, *Borago officinalis* L. showed notable antioxidant activity (and polyphenol content), supporting the established relationship between polyphenols and scavenging capacity [72]. *Sonchus oleraceus*, with its high chlorophyll content and moderate antioxidant activity, represents a species with balanced nutritional and bioactive compound profiles, as noted in previous studies [69]. *Cichorium intybus* demonstrated significant chlorophyll and flavonoid content, along with high antioxidant activity, confirming its reputation as a rich source of bioactive compounds [72].

## 4. Materials and Methods

### 4.1. Study Area

**Figure 10:**
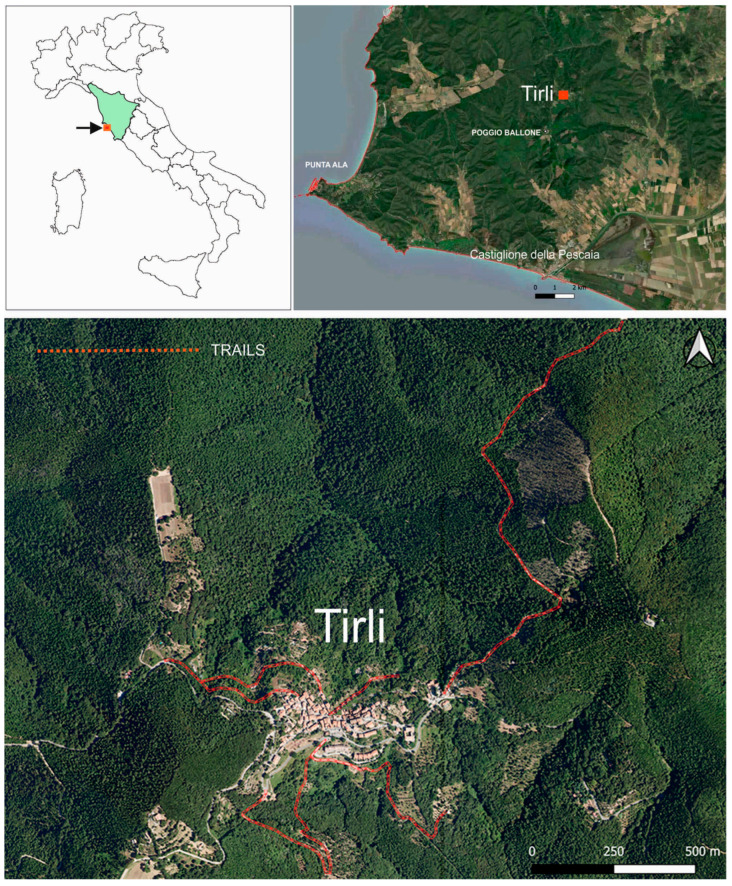
Location of the study area in Italy and Tuscany, including a close-up view of the study area and surrounding landscape, and trails (red lines) in the study area.

The study area (42°50′42.79″ N, 10°53’37.21″ E—WGS84) covers the surroundings of Tirli (Castiglione della Pescaia, Grosseto, Italy) (Figure 10), a small village with 255 inhabitants [73].

The research and floristic surveys were carried out along the main hiking trails connected to the village. These trails are frequently traversed by both locals and tourists and are notable for featuring structures of significant cultural and historical importance, such as old fountains. Altogether, the trails explored during the study extend for approximately 4.5 km, covering an approximate surface area of 27,000 m^2^.

**Figure 11:**
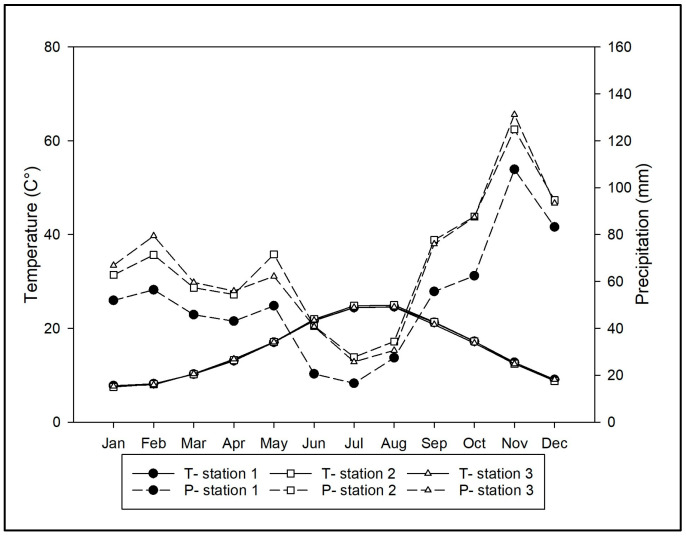
Ombrothermic diagram representation (see Study Area section for details).

Tirli is a hamlet within the municipality of Castiglione della Pescaia, part of the province of Grosseto in southern Tuscany. It lies around 10 km from the Tyrrhenian coast, at an elevation of 400 m above sea level, nestled within the hilly massif of Poggio Ballone (631 m a.s.l.). The vegetation cover of these hills, which are shaped by their geographic location, morphology, and altitudinal range, is influenced in its spatial distribution by both the altitudinal climatic gradient and thermal inversion [74]. The territory includes several forest types such as evergreen sclerophyllous forest, dominated by *Quercus ilex* L., either in pure stands or mixed with *Q. suber* L., and thermophilous deciduous forest, with evergreen sclerophyllous and deciduous species such as *Q. pubescens* Willd., *Q. cerris* L., *Q. frainetto* Ten., *Ostrya carpinifolia* Scop., *Robinia pseudoacacia* L., and *Castanea sativa* Mill. forest [75]. From a geological perspective, the predominant formations in the study area consist of Oligocene quartzofeldspathic sandstone [76], characterized by a high content of volcanic and carbonate lithic fragments [77]. The climate of the area can be included in the Mediterranean macrobioclimate, upper and lower meso-mediterranean thermotype, and sub-humid lower ombrotype [78]. To obtain a more detailed indication of the climatic characteristics, an ombrothermic diagram was created for the period 2003–2023 (Figure 11). Thermopluviometric data were extracted from three weather stations located within a 20 km radius of the study area: Casotto dei Pescatori (2 m a.s.l., station 1), Braccagni (40 m a.s.l., station 2), and Massa Marittima Valpiana (188 m a.s.l., station 3). The diagram highlights a summer drought period with a water deficit, particularly in the months of June and July. Across the investigated stations, the average annual precipitation is approximately 745 mm, while the mean annual temperature is close to 15.5 °C [79].

### 4.2. Data Collection and Analysis

Field activities were conducted during the spring seasons of the 2023–2024 period and, additionally, from September to November. The surveys involved detailed floristic research to document the WEPs’ relevance. Floristic entities were carefully selected, with particular attention given to the presence or absence of phytonutritional properties, following the criteria established in the literature [25,28,30,80,81,82,83]. The collected specimens were preserved in the herbarium at the Agricultural Botany Section of the Department of Agriculture, Food and Environment, University of Pisa. The species were identified according to Pignatti [54,84] and aligned with the updated nomenclature [24,85]. For the regional distribution and rarity of the species, the data obtained were assessed using information currently present on WikiplantbaseToscana [86], Portal of the Flora of Italy [87], and the last checklist of the vascular flora of the Tuscan Maremma [88]. To support their conservation, sustainable use, and integration into territorial planning, we analyzed and discussed the life-form, chorotype, and ecological indicator values attributed to them by Pignatti [54] and Ellenberg [57]. For each species, the local common name and information on the most used edible parts were reported, as gathered from the literature.

### 4.3. Biochemical Analysis

On-site interviews conducted for the B.E.T.A. project showed that 28 species from 20 families are already part of the local culinary tradition. These plants were consumed as food ingredients in salads, soups, and infusions. The 28 species were analyzed to assess the bioactive compounds associated with nutraceutical properties.

For each species, homogeneous samples were harvested from the internerval zone of the mid-lamina leaf area and pooled together. The leaves were washed under tap water, weighed (FW), and hot-air dried (60 °C for 48 h) until the dry weight (DW) was unchanged. The dried leaves were analyzed at the Plant Physiology Laboratory of the Department of Agricultural, Food and Environment, University of Pisa. The percentage of weight loss was calculated by the following formula: (FW − DW) × 100/FW.

The photosynthetic pigment (total chlorophylls and carotenoids) contents were determined using the Lichtenthaler method [89]. One hundred milligrams (100 mg) of dried leaves were homogenized with 2 mL of 100% methanol, and centrifuged for 10 min at 14,000 g. An aliquot of the supernatant was diluted 1:10, and the absorbance of the extracts at 665, 652, and 470 nm was measured using a UV-VIS spectrophotometer. The content of total chlorophyll and carotenoids was expressed as mg/g dry weight. The presented data are the means of three independent replicates.

For the determination of the phytochemical compounds (total polyphenols, total flavonoids, and antioxidant activity), 100 mg dried leaves were extracted with 2 mL of 70% methanol in an ice bath, maintained for 30 min, and centrifuged for 10 min at 14,000 g; the supernatant was recovered and used for further analysis. For the quantification of the total polyphenol content, the aliquots were submitted to the Folin–Ciocalteau method [90], measured at 765 nm, and expressed as mg of gallic acid equivalent (GAE) per g DW.

The total flavonoid content was measured at 510 nm and expressed in mg of catechin equivalent (CE) per g DW [91]. All the determinations were recorded using a UV–VIS spectrophotometer (SHIMADZU UV-1800, Shimadzu Corp., Kyoto, Japan).

The methanolic extracts were used to assess the antioxidant activity by the 2,2-diphenyl-1-picrylhydrazyl (DPPH) scavenging activity [92] and 2,2′-azino-bis (3-ethylbenzothiazoline-6-sulfonic acid (ABTS) antiradical activity [93]. Both assays were expressed in µmol Trolox equivalents (TE) per g DW.

Fifty milligrams (50 mg) of dried leaves were used for the extraction of the reducing sugars with 80% ethanol [94]. The samples were submitted to the 3,5-dinitrosalicylic acid (DNS) method [95]. The absorbance was measured at 540 nm, and the results were expressed in mg of glucose per g DW.

One hundred milligrams (100 mg) of dry leaves were powdered and mineralized with a solution of H_2_SO_4_ and H_2_O_2_ (15 min at 380 °C) and used to obtain the total organic nitrogen using the Kjeldahl method [96]. The crude protein amount was then estimated by multiplying the percentage of nitrogen by 6.25 as the conversion factor.

### 4.4. Statistical Analysis

To explore the interaction values between the different life-forms and chorological types, a contingency table was generated to examine their distribution, followed by a heatmap visualization to highlight patterns and interactions. This approach, which combines MCA and heatmap visualizations, offers a comprehensive analysis of the relationships between life-forms and their biogeographical distributions, providing an integrated perspective on plant species adaptation strategies to different environmental conditions. Multiple correspondence analysis (MCA) was applied to explore and visualize the interrelationships between the plant species and categorical variables organized in a binary matrix. In our analyses, we considered family, life-form, chorological type, and the plant parts used (roots, leaves, fruits, seeds, flowers). This statistical technique enables the synthesis of numerous qualitative variables into a limited number of dimensions, facilitating the identification of patterns and associations between observations and variable categories [97].

To identify the associations between variables, we computed the chi-square distance between various categories of variables. These associations were then visualized in Euclidean space, where variables positioned closely to each other at the periphery of the plot indicated positive associations, orthogonal variables indicated independence, and variables positioned 180 degrees apart signified negative associations. The proximity of variables to the plot’s periphery represented the strength of their association. This visualization facilitated a clearer understanding of the relationships between different observations and variables within our dataset. Furthermore, principal component analysis (PCA) was performed to investigate the relationships between the selected plants and their associated bioactive compounds. The results of the PCA were subsequently visualized using hierarchical cluster analysis based on Euclidean distance and Ward’s method, enabling the identification of patterns and clustering.

All the data analyses and visualization were performed using RStudio v. 2024.04.0 (Posit 2024) and R software v. 4.4.2 (R Core Team 2024).

## 5. Conclusions

The edible wild flora surveyed in the Tirli area reveal remarkable biodiversity, characterized by a broad representation of plant families and significant ecological adaptability.

The dominant families in this region include numerous edible species, well adapted to local environmental conditions and providing a variety of edible parts throughout the year. The richness of Tirli’s flora also reflects a deep cultural heritage, closely linked to the botanical and phytochemical traits of these plants.

This study underscores the diversity in the nutritional value of edible wild plants, identifying key species distinguished by their bioactive compound profiles and antioxidant properties. Future research should further investigate the potential applications of these species in dietary and therapeutic contexts, with a focus on optimizing their utilization as sources of health-promoting compounds.

## Data Availability

The complete floristic inventory, including information on the edible parts and bioactive compounds analyzed, is available in the Supplementary Materials.

## Supplementary Materials

The following supporting information can be downloaded at: https://www.mdpi.com/article/10.3390/plants14060976/s1, Table S1, Checklist of the wild edible plants (WEPs) listed in Tirli (Northern Maremma) and related edible parts; Table S2, Checklist of the wild edible plants (WEPs) listed in Tirli (Northern Maremma) and related Ellenberg indicator values; Table S3, Mean values (n = 4, ±SD) of bioactive compounds determined in selected species collected in Tirli area. Different letters indicate statistically significant differences (*p* < 0.05) based on Tukey’s Honest Significant Difference (HSD) test.

## Abbreviations

The following abbreviations are used in this manuscript:

ABTS	2,2′-azinobis (3-ethylbenzothiazoline-6-sulfonic acid)
CAR	Carotenoids
CE	Catechin equivalent
CHL*a*	Chlorophyll *a*
CHL*b*	Chlorophyll *b*
DNS	3,5-dinitrosalicylic acid
DPPH	2,2-diphenyl-1-picrylhydrazyl
DW	Dry weight
FW	Fresh weight
GAE	Gallic acid equivalent
GLU	Glucose
MCA	Multiple correspondence analysis
HCA	Hierarchical cluster analysis
PCA	Principal component analysis
TE	Trolox equivalent
WEPs	Wild edible plants
