# Supplementary material for: A Floristic Survey of Wild Edible Plants in Tuscan Maremma, Italy
Source: Plants (Basel). 2025 Mar 20;14(6):976. doi: 10.3390/plants14060976 (PMC11946747; doi:10.3390/plants14060976)
Supplement: Supplementary file 1 [file plants-14-00976-s001.zip › Supplementary Table S1.pdf]

Supplementary Table S1.

Checklist of the wild edible plants (WEPs) listed in Tirli (Northern Maremma) and related edible parts.

FAMILY/PLANT NAME	VULGAR NAME	LIFE FORM	CHOROTYPE	EDIBLE PARTS*
**AMARANTHACEAE**				
Amaranthus retroflexus L.	Amaranto	T scap	N-Americ	Leaves, seeds
Beta vulgaris L. subsp. *maritima* (L.) Arcang.	Bietola	H scap	Euri-Medit.	Leaves, flowers
Chenopodium album L. subsp. *album*	Spinacio selvatico, farinaccio	T scap	Subcosmop.	Leaves, seeds
**AMARYLLIDACEAE**				
Allium triquetrum L.	Aglietto, Aglio	G bulb	Steno-Medit.-Occid.	Roots, leaves, flowers
**APIACEAE**				
Anethum foeniculum L.	Finocchietto	H bienn	Euri-Medit.	Roots, leaves, seeds
Daucus carota L. subsp. *carota*	Carota selvatica	H scap	Centromedit.	Roots, leaves, seeds
Sanicula europaea L.	Erba fragolina	H ros	Paleotemp.	Leaves
Tordylium apulum L.	Ombrellino pugliese	T scap	Steno-Medit.	Leaves
**ASPARAGACEAE**				
Asparagus acutifolius L.	Asparago	G rhiz	Steno-Medit.	Shoots
Loncomelos pyrenaicum (L.) L.D.Hrouda subsp. *pyrenaicum*	Cipollaccio, Coda di scojattolo	G bulb	Euri-Medit.	Roots, flowers
Muscari comosum (L.) Mill.	Lampascione	G bulb	Euri-Medit.	Roots
Ruscus aculeatus L.	Pungitopo	P caesp	Euri-Medit.	Shoots
**ASTERACEAE**				
Anthemis arvensis L. subsp. *arvensis*	Camomilla senza odore	H scap	Subcosmop.	Flowers
Arctium lappa L.	Bardana, Lappa, Lappola.	H bienn	Eurasiat.	Roots, leaves, seeds
Bellis sylvestris Cirillo	Pratolina autunnale	H ros	Steno-Medit.	Leaves, flowers
Calendula arvensis (Vaill.) L.	Fior rancio de' campi	Ch suffr	Euri-Medit.	Flowers
Centaurea nigrescens Willd. subsp. *nigrescens*	Fiordaliso	H scap	Europ.	Leaves
Centaurea nigrescens Willd. subsp. *pinnatifida* (Fiori) Dostál	Fiordaliso	H scap	Endem. Ital.	Leaves
Cichorium intybus L.	Cicoria	H scap	Cosmop.	Roots, leaves
Crepis capillaris (L.) Wallr.	Radicchiella capillare	T scap	Centroeurop.	Leaves
Crepis leontodontoides All.	Radichiella, Crepide falso dente di leone	H ros	Medit.-Mont.	Leaves, flowers
Crepis sancta (L.) Bornm. subsp. *nemausensis* (P.Fourn.) Babc.	Radicchiella di Terrasanta	T scap	SE-Europ.	Leaves
Crepis setosa Haller f.	Radicchiella cotonosa	T scap	Euri-Medit.-Orient.	Leaves
Helichrysum italicum (Roth) G.Don subsp. *italicum*	Elicrizio	Ch suffr	S-Europ.	Leaves, flowers
Hieracium murorum L.	Sparviere dei muri	H scap	Eurosiber.	Leaves, flowers

Hypochaeris achyrophorus L.	Costolina annuale, Ipocheride annuale	T scap	Steno-Medit.	Leaves
Hypochaeris radicata L.	Ciabatte, Piattello	H ros	Europ.-Caucas.	Roots, leaves
Leucanthemum vulgare (Vaill.) Lam. subsp. *vulgare*	Bellide maggiore, Margherita	H scap	Eurosiber.	Leaves, flowers
Picris hieracioides L. subsp. *hieracioides*	Lattajola pungente	H scap	Eurasiat.	Leaves
Reichardia picroides (L.) Roth	Terracrepolo, Grattalingua, Caccialepre, Insalata di stecchi	H scap	Euri-Medit.	Leaves
Scolymus hispanicus L. subsp. *hispanicus*	Barba gentile	H bienn	Euri-Medit.	Roots, leaves, flowers
Silybum marianum (L.) Gaertn.	Cardo asinino, Cardo Mariano	H bienn	Medit.-Turan.	Leaves, flowers, seeds
Sonchus arvensis L. subsp. *arvensis*	Grespino dei campi	H scap	Subcosmop.	Roots, leaves, flowers
Sonchus asper (L.) Hill subsp. *asper*	Grespino spinoso	T scap	Subcosmop.	Roots, leaves, flowers
Sonchus oleraceus L.	Cicerbita	T scap	Subcosmop.	Roots, leaves, flowers
Taraxacum F.H.Wigg. sect. t*araxacum*	Tarassaco, Dente di leone	H ros	Circumbor.	Roots, leaves, flowers
Urospermum dalechampii (L.) Scop. ex F.W.Schmidt	Terracrepolo, Insalata di campo	H scap	Steno-Medit.	Roots, leaves, flowers
Urospermum picroides (L.) Scop. ex F.W.Schmidt	Boccione minore	T scap	Euri-Medit.	Roots, leaves, flowers
**BETULACEAE**				
Corylus avellana L.	Nocciolo	P caesp	Europ.-Caucas.	Fruits
**BORAGINACEAE**				
Borago officinalis L.	Borraggine	T scap	Euri-Medit.	Leaves, flowers
Echium italicum L. subsp. *italicum*	Echio, Lingua	H bienn	Euri-Medit.	Leaves
**BRASSICACEAE**				
Alliaria petiolata (M. Bieb.) Cavara & Grande	Alliaria	H bienn	Paleotemp.	Roots, leaves, flowers, seeds
Cardamine hirsuta L.	Billeri	T scap	Cosmop.	Leaves, flowers
Diplotaxis tenuifolia (L.) DC.	Rucola	H scap	Submedit.	Leaves
Lunaria annua L.	Erba argentina, Erba lunaria	H scap	S-Europ.	Roots, leaves, seeds
Sinapis arvensis L. subsp. *arvensis*	Senape	T scap	Steno-Medit.	Leaves, flowers, seeds
Sisymbrium officinale (L.) Scop.	Erba cornacchia	T scap	Subcosmop.	Leaves, flowers, seeds
**CAMPANULACEAE**				
Campanula rapunculus L.	Raponzolo, Raperonzolo	H bienn	Paleotemp.	Roots, leaves
**CARYOPHYLLACEAE**				
Silene latifolia Poir.	Strigolo	H scap	Steno-Medit.	Roots, leaves, flowers
Stellaria media (L.) Vill.	Centocchio	T rept	Cosmop.	Shoots
**CRASSULACEAE**				
Umbilicus rupestris (Salisb.) Dandy	Ombellico di Venere, Palanconi	G bulb	Medit.-Atl.	Leaves
**CYPERACEAE**				
Cyperus rotundus L.	Cipero rotondo	G rhiz	Subcosmop.	Roots

**DIOSCOREACEAE**				
Dioscorea communis (L.) Caddick & Wilkin	Tamaro, Uva tamina, Vite nera	G rad	Euri-Medit.	Shoots
**DIPSACACEAE**				
Knautia integrifolia (L.) Bertol. subsp. *integrifolia*	Ambretta annuale	T scap	Euri-Medit.	Leaves
Scabiosa columbaria L. subsp. *columbaria*	Vedovella, Vedovina selvatica	H scap	Eurasiat.	Leaves, flowers
Sixalix atropurpurea (L.) Greuter & Burdet	Scabiosa atropurpurea	H scap	Steno-Medit.	Leaves
**ERICACEAE**				
Arbutus unedo L.	Corbezzolo	P caesp	Steno-Medit.	Fruits
**EUPHORBIACEAE**				
Mercurialis annua L.	Mercorella	T scap	Paleotemp.	Leaves
**FABACEAE**				
Lathyrus clymenum L.	Veccia selvatica	T scap	Steno-Medit.	Seeds
Lathyrus oleraceus Lam. subsp. *biflorus* (Raf.) H.Schaef., Coulot & Rabaute	Pisello	T scap	Medit.-Turan.	Seeds
Robinia pseudoacacia L.	Acacia	P caesp	N-Americ.	Flowers
Trifolium campestre Schreb.	Pratolina salvatica	T scap	Paleotemp.	Leaves, flowers
Trifolium repens L.	Trifoglio bianco	H rept	Subcosmop.	Leaves, flowers
Vicia cracca L.	Veccia cracca	H scap	Eurasiat.	Leaves, flowers, seeds
Vicia sativa L.	Veccia dolce, Veccia comune	T scap	Subcosmop.	Leaves, flowers
**FAGACEAE**				
Castanea sativa Mill.	Castagno	P scap	SE-Europ.	Fruits
Quercus ilex L.	Leccio	P scap	Steno-Medit.	Fruits
**GENTIANACEAE**				
Centaurium erythraea Rafn subsp. *erythraea*	Cacciafebbre	H bienn	Paleotemp.	Leaves, flowers
**HYPERICACEAE**				
Hypericum perforatum L. subsp. *veronense* (Schrank) Ces.	Iperico, Erba di San Giovanni	H caesp	Medit.-Turan.	Leaves, flowers
**LAMIACEAE**				
Clinopodium nepeta (L.) Kuntze subsp. *nepeta*	Inpitella, Mentuccia	H scap	Medit.-Turan.	Leaves, flowers
Clinopodium vulgare L. subsp. *vulgare*	Clinopodio dei boschi	H scap	Circumbor.	Leaves, flowers
Lamium maculatum L.	Falsa ortica macchiata	H scap	Eurasiat.	Leaves, flowers, shoots
Lavandula stoechas L. subsp. *stoechas*	Lavanda	NP	Steno-Medit.	Leaves, flowers
Melissa officinalis L. subsp. *officinalis*	Melissa	H scap	Euri-Medit.	Leaves
Mentha suaveolens Ehrh. subsp. *suaveolens*	Menta	H scap	Euri-Medit.	Leaves, flowers
Origanum vulgare L. subsp. *vulgare*	Origano	H scap	Eurasiat.	Leaves, flowers
Prunella vulgaris L. subsp. *vulgaris*	Brunella	H scap	Circumbor.	Leaves, flowers
**LAURACEAE**				
Laurus nobilis L.	Alloro	P caesp	Steno-Medit.	Leaves, fruits
**LINACEAE**				
Linum usitatissimum L. subsp. *angustifolium* (Huds.) Thell.	Lino	T scap	Euri-Medit.	Seeds
**MALVACEAE**				
Malva sylvestris L.	Malva	H scap	Subcosmop.	Roots, leaves
**MORACEAE**				
Ficus carica L.	Fico	P scap	Medit.-Turan.	Fruits, leaves
**MYRTACEAE**				

Myrtus communis L.	Mirto	P scap	Steno-Medit.	Leaves, fruits
**OLEACEAE**				
Fraxinus ornus L. subsp. *Ornus*	Orniello	P scap	Europ.-Caucas.	Fruits, leaves
Olea europaea L.	Olivo	P scap	Steno-Medit.	Fruits
**OXALIDACEAE**				
Oxalis articulata Savigny	Acetosella rizomatosa	G rhiz	S-Americ.	Leaves, flowers
**PAPAVERACEAE**				
Papaver rhoeas L. subsp. *rhoeas*	Papavero comune	T scap	E-Medit.	Roots, leaves, flowers, seeds
Papaver somniferum L.	Papavero da oppio	T scap	Subcosmop.	Leaves, flowers, seeds
**PLANTAGINACEAE**				
Linaria vulgaris Mill. subsp. *vulgaris*	Linarita, Ramerito selvatico	H scap	Eurasiat.	Flowers
Plantago coronopus L.	Barba del prete, Barba di cappuccino	H ros	Euri-Medit.	Leaves, flowers, roots
Plantago lanceolata L.	Piantaggine	H ros	Cosmop.	Leaves, seed
Plantago major L.	Piantaggine maggiore	H ros	Subcosmop.	Leaves, seed, flowers
Veronica polita Fr.	Veronica lucida	T scap	Subcosmop.	Leaves, flowers
**POACEAE**				
Avena barbata Pott ex Link	Avena barbata	T scap	Medit.-Turan.	Seeds
Cynodon dactylon (L.) Pers.	Gramigna rossa	G rhiz	Subcosmop.	Roots, leaves
Elymus repens (L.) Gould subsp. *repens*	Gramigna	G rhiz	Circumbor.	Roots, leaves, seeds
**POLYGONACEAE**				
Rumex acetosella L. subsp. *acetosella*	Acetosa, Acetosella	H scap	Subcosmop.	Leaves
Rumex patientia L. subsp. *patientia*	Romice, Rombice, Pazienza	H scap	Europ.	Leaves
Rumex pulcher L. subsp. *pulcher*	Romice cavolaccio	H scap	Subcosmop.	Leaves
**POLYPODIACEAE**				
Polypodium vulgare L.	Polipodio	H ros	Paleotemp.	Roots, leaves
**PORTULACACEAE**				
Portulaca oleracea L.	Portulaca	T scap	Subcosmop.	Leaves
**PRIMULACEAE**				
Primula vulgaris Huds. subsp. *vulgaris*	Primula, Primavera, Occhio di civetta	H ros	Europ.-Caucas.	Leaves, flowers
**RANUNCULACEAE**				
Clematis vitalba L.	Vitalba, Vezzadri, Vizzadro	P lian	Europ.-Caucas.	Shoots
**ROSACEAE**				
Agrimonia eupatoria L. subsp. *eupatoria*	Erba di San Guglielmo	H scap	Subcosmop.	Leaves
Potentilla recta L. subsp. *recta*	Cinquefoglia diritta	H scap	NE-Medit.	Leaves
Potentilla reptans L.	Cinquefoglia comune	H ros	Subcosmop.	Leaves
Poterium sanguisorba L. subsp. *sanguisorba*	Salvastrella, Pimpinella	H scap	Paleotemp.	Leaves
Prunus avium (L.) L.	Ciliegio	P scap	Europ.-Caucas.	Fruits
Prunus spinosa L. subsp. *spinosa*	Prugnolo spinoso	P caesp	Europ.-Caucas.	Fruits, flowers
Pyrus communis L.	Pero	P scap	Europ.-Caucas.	Fruits
Rosa canina L.	Rosa canina	NP	Paleotemp.	Fruits, flowers

Rubus ulmifolius Schott	Germoglio di Rovo, Mora	P caesp	Euri-Medit.	Fruits, shoots
Sorbus domestica L.	Sorbe	P scap	Euri-Medit.	Leaves, fruits
**RUBIACEAE**				
Galium verum L. subsp. *verum*	Caglio zolfino	H scap	Eurasiat.	Leaves, flowers
Rubia peregrina L.	Robbia	P lian	Medit.-Macarones.	Roots, fruits
**SMILACACEAE**				
Smilax aspera L.	Salsapariglia nostrana	P lian	Paleosubtrop.	Shoots, roots
**TYPHACEAE**				
Typha latifolia L.	Tifa, Stiancia, Schiancia	G rhiz	Cosmop.	Roots, leaves
**ULMACEAE**				
Ulmus minor Mill. subsp. *minor*	Olmo	P scap	Europ.-Caucas.	Leaves, fruits
**URTICACEAE**				
Parietaria judaica L.	Muraiola	H scap	W-Medit.-Macarones.	Leaves
Parietaria officinalis L.	Vetriola	H scap	Medit.-Turan.	Leaves
Urtica dioica L.	Ortica	H scap	Subcosmop.	Leaves
**VALERIANACEAE**				
Centranthus ruber (L.) DC. subsp. *ruber*	Valeriana rossa	Ch suffr	Steno-Medit.	Leaves, flowers, shoots
Valerianella locusta (L.) Laterr.	Valerianella	T scap	Euri-Medit.	Leaves, flowers
**VIBURNACEAE**				
Sambucus nigra L.	Sambuco	P caesp	Europ.-Caucas.	Flowers, fruits
**VIOLACEAE**				
Viola alba Besser subsp. *dehnhardtii* (Ten.) W.Becker	Viola bianca	H ros	Steno-Medit.	Leaves, flowers
Viola arvensis Murray	Viola dei campi	T scap	Eurasiat.	Leaves, flowers
Viola odorata L.	Viola mammola	H ros	Euri-Medit.	Leaves, flowers
**VITACEAE**				
Vitis vinifera L.	Vite	P lian	Medit.-Turan.	Leaves, fruits, seeds
**ZYGOPHYLLACEAE**				
Tribulus terrestris L.	Tribolo	T rept	Cosmop.	Seeds

* The information regarding the edibility of the organs listed for each species has been entirely derived from the references cited below:

- A database of edible and otherwise useful plants.
http://www.pfaf.org/user/Default.aspx
- A database of edible plants, with information on their nutritional value.
https://fms.cmsvr.com/fmi/webd/Food_Plants_World
- A database of the Italian flora, with information on properties and uses of plants.
https://www.actaplantarum.org/
- An ongoing collaborative space for the exchange of information on useful plants.
https://uses.plantnet-project.org/en/Main_Page
- Database of wild edible plants.
https://phytoalimurgia.it/
- Information on wild edible plants, with information on the edibility of plants.
https://www.pianteinnovative.it/
- Paura, B.; Di Marzio, P.; Salerno, G.; Brugiapaglia, E.; Bufano, A. Design a Database of Italian Vascular Alimurgic Flora (AlimurgITA): Preliminary Results. *Plants* **2021**, *10*, 743.
- Portale della Flora d'Italia - Portal to the Flora of Italy. 2024.3.
http://dryades.units.it/floritaly/
