# Supplementary material for: A Floristic Survey of Wild Edible Plants in Tuscan Maremma, Italy
Source: Plants (Basel). 2025 Mar 20;14(6):976. doi: 10.3390/plants14060976 (PMC11946747; doi:10.3390/plants14060976)
Supplement: Supplementary file 1 [file plants-14-00976-s001.zip › Supplementary Table S2.pdf]

Supplementary Table S2.

Checklist of the wild edible plants (WEPs) listed in Tirli (Northern Maremma) and related Ellenberg indicator values.
T = Temperature; **L** = Light; **R** = Soil Reaction; **C** = Continentality; **N** = Soil Nutrients; **U** = Soil Moisture; **S** = Salinity.

Abbreviations:
X – broad-spectrum species
0 – insufficient information

TAXA	ELLENBERG INDICATOR VALUES						
	L	T	C	U	R	N	S
Agrimonia eupatoria L. subsp. *eupatoria*	7	6	5	4	8	4	0
Alliaria petiolata (M. Bieb.) Cavara & Grande	5	6	5	5	7	9	0
Allium triquetrum L.	6	9	4	4	4	7	0
Amaranthus retroflexus L.	9	9	7	4	X	9	0
Anethum foeniculum L.	9	8	5	3	7	7	0
Anthemis arvensis L. subsp. *arvensis*	7	6	4	4	3	6	0
Arbutus unedo L.	11	9	4	3	4	2	0
Arctium lappa L.	9	5	5	5	7	9	0
Asparagus acutifolius L.	6	9	4	2	5	5	0
Avena barbata Pott ex Link	8	8	5	3	7	2	0
Bellis sylvestris Cirillo	5	8	4	3	3	3	0
Beta vulgaris L. subsp. *maritima* (L.) Arcang.	11	7	5	6	6	5	1
Borago officinalis L.	7	8	5	3	5	5	0
Calendula arvensis (Vaill.) L.	7	8	5	3	8	5	0
Campanula rapunculus L.	7	7	5	4	6	4	0
Cardamine hirsuta L.	7	8	5	3	5	4	0
Castanea sativa Mill.	5	8	6	X	4	X	0
Centaurea nigrescens Willd. subsp. *nigrescens*	7	6	5	4	5	4	0
Centaurea nigrescens Willd. subsp. *pinnatifida* (Fiori) Dostál	7	6	5	4	5	4	0
Centaurium erythraea Rafn subsp. *erythraea*	8	6	5	5	6	X	0
Centranthus ruber (L.) DC. subsp. *ruber*	6	8	4	2	X	1	0
Chenopodium album L. subsp. *album*	7	7	5	4	5	7	0
Cichorium intybus L.	9	6	5	3	8	5	0
Clematis vitalba L.	7	7	4	5	7	7	0
Clinopodium nepeta (L.) Kuntze subsp. *nepeta*	5	7	5	3	9	3	0
Clinopodium vulgare L. subsp. *vulgare*	7	5	4	4	7	3	0
Corylus avellana L.	6	5	4	5	5	8	0
Crepis capillaris (L.) Wallr.	7	6	5	4	5	3	0

Crepis leontodontoides All.	5	8	4	4	3	7	0
Crepis sancta (L.) Bornm. subsp. *nemausensis* (P.Fourn.) Babc.	11	9	6	2	X	2	0
Crepis setosa Haller f.	11	9	6	2	X	2	0
Cynodon dactylon (L.) Pers.	8	8	5	4	X	4	0
Cyperus rotundus L.	8	10	5	6	8	5	0
Daucus carota L. subsp. *carota*	8	6	5	4	5	4	0
Dioscorea communis (L.) Caddick & Wilkin	5	7	5	5	8	6	0
Diplotaxis tenuifolia (L.) DC.	8	7	5	4	6	5	0
Echium italicum L. subsp. *italicum*	11	8	5	3	3	4	0
Elymus repens (L.) Gould subsp. *repens*	7	X	7	5	X	8	0
Ficus carica L.	7	8	6	X	5	X	0
Fraxinus ornus L. subsp. *ornus*	5	8	6	3	8	3	0
Galium verum L. subsp. v*erum*	7	6	6	4	7	3	0
Helichrysum italicum (Roth) G.Don subsp. *italicum*	8	8	5	4	3	2	0
Hieracium murorum L.	4	X	4	5	5	X	0
Hypericum perforatum L. subsp. *veronense* (Schrank) Ces.	7	8	6	X	X	X	0
Hypochaeris achyrophorus L.	11	9	4	2	X	2	0
Hypochaeris radicata L.	9	8	4	2	X	1	0
Knautia integrifolia (L.) Bertol. subsp. *integrifolia*	7	8	5	3	3	2	0
Lamium maculatum L.	7	7	5	4	5	4	0
Lathyrus clymenum L.	7	8	4	4	3	3	0
Lathyrus oleraceus Lam. subsp. *biflorus* (Raf.) H.Schaef., Coulot & Rabaute	9	9	4	3	4	3	0
Laurus nobilis L.	2	7	4	8	4	6	0
Lavandula stoechas L. subsp. *stoechas*	11	9	4	2	1	1	0
Leucanthemum vulgare (Vaill.) Lam. subsp. *vulgare*	7	X	4	4	X	3	0
Linaria vulgaris Mill. subsp. *vulgaris*	8	5	5	3	7	3	0
Linum usitatissimum L. subsp. *angustifolium* (Huds.) Thell.	9	7	5	4	3	3	0
Loncomelos pyrenaicum (L.) L.D.Hrouda subsp. *pyrenaicum*	5	5	5	6	5	5	0
Lunaria annua L.	4	6	6	6	7	6	0
Malva sylvestris L.	8	6	4	4	X	8	0
Melissa officinalis subsp. *officinalis* L.	6	7	5	4	6	4	0
Mentha suaveolens Ehrh. subsp. *suaveolens*	7	8	5	8	7	6	0
Mercurialis annua L.	7	7	5	4	7	8	0
Muscari comosum (L.) Mill.	7	8	5	3	7	0	0
Myrtus communis L.	8	9	4	3	5	2	0

Olea europaea L.	11	10	4	1	X	2	0
Origanum vulgare L. subsp. *vulgare*	7	6	5	3	X	3	0
Oxalis articulata Savigny	X	X	X	X	X	X	X
Papaver rhoeas L. subsp. *rhoeas*	6	6	5	5	7	X	0
Papaver somniferum L.	X	X	X	X	X	X	X
Parietaria judaica L.	7	8	5	3	X	6	0
Parietaria officinalis L.	4	8	4	5	7	7	0
Picris hieracioides L. subsp. *hieracioides*	8	X	5	4	8	4	0
Plantago coronopus L.	8	7	5	7	7	4	0
Plantago lanceolata L.	6	7	5	X	X	X	0
Plantago major L.	8	X	X	5	X	7	0
Polypodium vulgare L.	5	X	4	X	2	X	0
Portulaca oleracea L.	7	8	5	4	7	7	0
Potentilla recta L. subsp. *recta*	9	7	6	3	7	3	0
Potentilla reptans L.	6	6	5	6	7	5	0
Poterium sanguisorba L. subsp. *sanguisorba*	7	6	5	3	8	2	0
Primula vulgaris Huds. subsp. *vulgaris*	6	5	4	5	7	5	0
Prunella vulgaris L. subsp. *vulgaris*	7	6	4	6	4	X	0
Prunus avium (L.) L.	4	5	6	5	7	5	0
Prunus spinosa L. subsp. *spinosa*	7	5	5	X	X	X	0
Pyrus communis L.	7	7	5	5	5	5	0
Quercus ilex L.	2	9	4	3	X	X	0
Reichardia picroides (L.) Roth	7	8	4	3	6	2	0
Robinia pseudoacacia L.	5	7	5	4	X	8	0
Rosa canina L.	8	5	5	4	X	X	0
Rubia peregrina L.	5	9	4	4	5	3	0
Rubus ulmifolius Schott	5	8	5	4	5	8	0
Rumex acetosella L. subsp. acetosella	8	5	5	5	1	2	0
Rumex patientia L. subsp. patientia	7	6	4	3	6	7	0
Rumex pulcher L. subsp. *pulcher*	8	8	5	2	6	9	0
Ruscus aculeatus L.	4	8	5	4	5	5	0
Sambucus nigra L.	7	5	4	5	X	9	0
Sanicula europaea L.	4	5	5	5	8	6	0
Scabiosa columbaria L. subsp. *columbaria*	8	5	5	4	8	2	0
Scolymus hispanicus L. subsp. *hispanicus*	11	8	5	3	X	2	0

Silene latifolia Poir.	6	9	4	3	4	2	0
Silybum marianum (L.) Gaertn.	11	10	6	3	5	7	0
Sinapis arvensis L. subsp. *arvensis*	7	5	4	X	8	6	0
Sisymbrium officinale (L.) Scop.	8	6	5	4	X	7	0
Sixalix atropurpurea (L.) Greuter & Burdet	6	8	4	3	X	2	0
Smilax aspera L.	6	10	4	2	5	3	0
Sonchus arvensis L. subsp. *arvensis*	7	5	X	5	7	X	0
Sonchus asper (L.) Hill subsp. *asper*	7	5	X	4	7	7	0
Sonchus oleraceus L.	7	5	X	4	8	8	0
Sorbus domestica L.	4	7	5	3	8	3	0
Stellaria media (L.) Vill.	6	X	X	4	7	8	0
Taraxacum F.H.Wigg. sect. *taraxacum*	7	X	X	5	X	7	0
Tordylium apulum L.	11	9	4	2	X	2	0
Tribulus terrestris L.	8	8	6	2	5	3	0
Trifolium campestre Schreb.	8	5	5	4	X	3	0
Trifolium repens L.	8	X	X	X	X	7	0
Typha latifolia L.	8	6	5	10	X	8	0
Ulmus minor Mill. subsp. *minor*	5	7	5	X	8	X	0
Umbilicus rupestris (Salisb.) Dandy	5	8	4	3	X	3	0
Urospermum dalechampii (L.) Scop. ex F.W.Schmidt	8	8	5	3	X	3	0
Urospermum picroides (L.) Scop. ex F.W.Schmidt	11	9	5	2	X	2	0
Urtica dioica L.	X	X	X	6	X	8	0
Valerianella locusta (L.) Laterr.	7	5	5	5	7	X	0
Veronica polita Fr.	5	6	5	4	8	7	0
Vicia cracca L.	7	X	X	5	X	X	0
Vicia sativa L.	5	5	6	X	X	X	0
Viola alba Besser subsp. *dehnhardtii* (Ten.) W.Becker	5	8	5	5	7	6	0
Viola arvensis Murray	5	5	5	X	X	X	0
Viola odorata L.	5	6	5	5	X	8	0
Vitis vinifera L.	6	8	5	6	8	6	0
