# Supplementary material for: A Floristic Survey of Wild Edible Plants in Tuscan Maremma, Italy
Source: Plants (Basel). 2025 Mar 20;14(6):976. doi: 10.3390/plants14060976 (PMC11946747; doi:10.3390/plants14060976)
Supplement: Supplementary file 1 [file plants-14-00976-s001.zip › Supplementary Table S3.pdf]

Supplementary Table S3.

Mean values (n= 4, ± SD) of bioactive compounds determined in selected species collected in Tirli area. Different letters indicate statistically significant differences (p<0.05) based on Tukey's Honest Significant Difference (HSD) test. CHLa/CHLb: Chlorophyll a / Chlorophyll b; ABTS: 2,2'-azino-bis (3-ethylbenzothiazoline-6-sulfonic acid); DPPH: 2,2-diphenyl-1-picrylhydrazyl.

TAXA	TOTAL CHLOROPHYLL mg/g DW	CHLa/CHLb	TOTAL CAROTENOIDS mg/g DW	TOTAL POLYPHENOLS mg GAE /g DW	TOTAL FLAVONOIDS mg CE/g DW	ABTS assay mM TE/g DW	DPPH assay mM TE/g DW	SOLUBLE SUGAR mg GLU/g	PROTEIN CONTENT %
Alliaria petiolata (M. Bieb.) Cavara & Grande	10.11 ± 1.48 ^{lnop}	2.8 ± 0.25 ^{ef}	1.86 ± 0.17 ^{abcd}	35.94 ± 0.42 ^{lm}	10.71 ± 0.5 ^{o}	5.51 ± 0.26 ^{klm}	5.27 ± 0.11 ^{dn}	72.91 ± 1.52 ^{p}	30.64 ± 1.01 ^{gp}
Allium triquetrum L.	6.61 ± 0.42 ^{abcd}	2.35 ± 0.47 ^{cdef}	1.03 ± 0.23 ^{abc}	20.13 ± 0.53 ^{a}	4.78 ± 0.18 ^{a}	3.03 ± 0.34 ^{a}	1.17 ± 0.08 ^{a}	33.94 ± 2.3 ^{a}	17.68 ± 0.26 ^{abc}
Anethum foeniculum L.	5.07 ± 0.47 ^{acef}	2.52 ± 0.21 ^{def}	0.91 ± 0.07 ^{abe}	45.95 ± 0.29 ^{b}	32.82 ± 0.76 ^{bc}	8.2 ± 0.4 ^{bc}	10.99 ± 0.17 ^{bc}	46.35 ± 0.88 ^{b}	25.05 ± 0.76 ^{def}
Beta vulgaris L. subsp. *maritima* (L.) Arcang.	5.8 ± 0.78 ^{abce}	2.04 ± 0.43 ^{cdef}	0.88 ± 0.13 ^{bef}	30.1 ± 0.09 ^{cde}	10.19 ± 0.02 ^{o}	5.06 ± 0.19 ^{lm}	4.61 ± 0.13 ^{d}	31.03 ± 0.88 ^{a}	27.57 ± 1.53 ^{dg}
Borago officinalis L.	4 ± 0.04 ^{cefg}	2.09 ± 0.21 ^{cdef}	0.61 ± 0.07 ^{ghi}	82.68 ± 0.09 ^{f}	78.06 ± 0.65 ^{d}	12.32 ± 0.01 ^{de}	6.19 ± 0.27 ^{e}	62.49 ± 1.41 ^{cd}	19.38 ± 0.05 ^{abh}
Campanula rapunculus L.	15.51 ± 0.18 ^{h}	2.17 ± 0.02 ^{cdef}	2.36 ± 0.01 ^{fj}	34.62 ± 0.69 ^{elm}	23.67 ± 0.48 ^{ef}	8.91 ± 0.2 ^{b}	9.1 ± 0.05 ^{fgh}	54.36 ± 0.61 ^{ef}	18.24 ± 0.07 ^{ab}
Chenopodium album L. subsp. *album*	6.26 ± 0.09 ^{abcd}	2.36 ± 0.01 ^{cdef}	1.47 ± 0.02 ^{dklm}	16.49 ± 0.13 ^{a}	9.81 ± 0.2 ^{o}	3.53 ± 0.26 ^{af}	7.96 ± 0.07 ^{ij}	42.39 ± 0.9 ^{bgh}	21.08 ± 0.25 ^{ahi}
Cichorium intybus L.	15.65 ± 0.01 ^{h}	2 ± 0.01 ^{bcdef}	1.92 ± 0.01 ^{efj}	31.14 ± 0.18 ^{cdel}	21.11 ± 0.33 ^{e}	6.19 ± 0.29 ^{hkl}	9.44 ± 0.01 ^{fg}	64.94 ± 1.34 ^{cdi}	14.46 ± 0.78 ^{cjkl}
Clematis vitalba L.	12.37 ± 0.03 ^{jkn}	2.1 ± 0 ^{cdef}	2.17 ± 0 ^{g}	37.99 ± 0.02 ^{m}	28.98 ± 0.6 ^{bg}	7.25 ± 0.17 ^{cgh}	8.66 ± 0.01 ^{h}	71.55 ± 0.64 ^{kp}	4.78 ± NA ^{m}
Clinopodium nepeta (L.) Kuntze subsp. *nepeta*	8.89 ± 0.18 ^{dlop}	2.1 ± 0.01 ^{cdef}	1.46 ± 0.03 ^{dklm}	50.23 ± 0 ^{b}	34.28 ± 0.06 ^{ch}	11.17 ± 0.02 ^{di}	11.43 ± 0.07 ^{bk}	57.47 ± 0.17^{ej}	18.33 ± 0.39 ^{ab}
Clinopodium vulgare L. subsp. *vulgare*	2.69 ± 0.03 ^{fg}	0.87 ± 0 ^{a}	0.28 ± 0.01 ^{j}	137.14 ± 1.3 ^{g}	134.16 ± 1.14 ^{i}	11.5 ± 0.08 ^{dei}	10.68 ± 0.02 ^{cl}	61.09 ± 0.04 ^{cj}	11.28 ± NA ^{jkn}
Crepis leontodontoides All.	12.83 ± 0.13 ^{ijk}	1.93 ± 0 ^{abcdef}	1.87 ± 0.02 ^{fj}	71.78 ± 0.31 ^{h}	67.48 ± 0.66 ^{j}	12.01 ± 0.48 ^{de}	8.62 ± 0.11 ^{hi}	67.55 ± 0.28 ^{ik}	16.92 ± NA ^{abckl}
Hypericum perforatum L. subsp. *veronense* (Schrank) Ces.	8.37 ± 0.02 ^{bdlp}	2.29 ± 0.03 ^{cdef}	1.46 ± 0.02 ^{abe}	111.23 ± 0.77 ^{i}	73.97 ± 1.75 ^{dk}	11.44 ± 0.41 ^{dei}	11.61 ± 0.08 ^{bk}	97.82 ± 1.65 ^{l}	16.24 ± 0.81 ^{bcl}
Laurus nobilis L.	3.36 ± 0.05 ^{efg}	1.31 ± 0.01 ^{abc}	0.47 ± 0.01 ^{acdk}	80.82 ± 0.37 ^{f}	45.64 ± 1.09 ^{l}	12.7 ± 0.09 ^{ej}	11.89 ± 0.1 ^{k}	130.47 ± 0.15 ^{m}	13.38 ± 0.43 ^{jkl}
Lunaria annua L.	15.48 ± 1.53 ^{hi}	2.4 ± 0.52 ^{def}	2.67 ± 0.13 ^{abc}	27.71 ± 0.76 ^{c}	11.28 ± 0.1 ^{o}	4.39 ± 0.11 ^{fm}	5.31 ± 0.05 ^{n}	39.41 ± 0.11 ^{gn}	26.07 ± 0.83 ^{de}
Malva sylvestris L.	11.17 ± 0.33 ^{kno}	2.32 ± 0.01 ^{cdef}	1.44 ± 0.04 ^{acdk}	18.48 ± 0.43 ^{a}	10.15 ± 0.95 ^{o}	3.13 ± 0.03 ^{af}	4.98 ± 0.2 ^{dn}	39.93 ± 1.23 ^{ghn}	37.59 ± 0.1 ^{o}
Melissa officinalis L. subsp. *officinalis*	14.39 ± 0.16 ^{hij}	1.75 ± 0.02 ^{abcde}	1.94 ± 0.03 ^{gh}	74.28 ± 0.35 ^{h}	73.37 ± 0.01 ^{k}	11.86 ± 0.01 ^{de}	10.63 ± 0.14 ^{cl}	67.17 ± 0.15^{dik}	22.21 ± 0.23 ^{fhi}
Myrtus communis L.	1.73 ± 0.06 ^{g}	0.93 ± 0.03 ^{ab}	0.13 ± 0.01 ^{cdkl}	146.81 ± 0.73 ^{j}	13.73 ± 0.12 ^{no}	11.27 ± 0.04 ^{di}	10.68 ± 0.15 ^{cl}	81.49 ± 0.18 ^{o}	7.8 ± 0.04 ^{mn}

Plantago coronopus L.	1.99 ± 0.2 ^{g}	1.79 ± 0.44 ^{abcdef}	0.31 ± 0.05 ^{klm}	109.83 ± 4.37 ^{i}	106.42 ± 2.81 ^{m}	11.52 ± 0.29 ^{dei}	7.48 ± 0.25 ^{j}	78.8 ± 0.44 ^{o}	7.17 ± 0.11 ^{mn}
Portulaca oleracea L.	3.21 ± 0.09 ^{efg}	1.68 ± 0.03 ^{abcd}	0.84 ± 0.03 ^{no}	26.81 ± 0.2 ^{c}	16.5 ± 0.19 ^{n}	5.18 ± 0.08 ^{lm}	9.28 ± 0.07 ^{fgh}	39.44 ± 0.03 ^{gn}	23.59 ± 0.02 ^{efi}
Poterium sanguisorba L. subsp. *sanguisorba*	6.99 ± 0.06 ^{abd}	1.88 ± 0.01 ^{abcde}	1.17 ± 0.02 ^{hin}	128.63 ± 0.95 ^{k}	38.65 ± 0.05 ^{h}	11.98 ± 0.03 ^{de}	9.02 ± 0.01 ^{gh}	79.63 ± 0.07 ^{o}	13.22 ± 0.1 ^{jkl}
Reichardia picroides (L.) Roth	7.53 ± 0.12 ^{abdl}	1.84 ± 0.02 ^{abcde}	1.62 ± 0.04 ^{dklm}	84.92 ± 0.21 ^{f}	74.4 ± 0.27 ^{dk}	13.71 ± 0.04 ^{j}	9.76 ± 0.03 ^{fm}	72.73 ± 0.49 ^{p}	11.78 ± 0.24 ^{j}
Rumex acetosella L. subsp. *acetosella*	7.31 ± 0.2 ^{abd}	1.56 ± 0.01 ^{abcd}	0.92 ± 0.02 ^{mno}	28.94 ± 0.18 ^{cd}	21.54 ± 0.5 ^{e}	8.9 ± 0.21 ^{b}	10.33 ± 0.02 ^{lm}	60.87 ± 1.08 ^{cj}	29.55 ± NA ^{dgp}
Sambucus nigra L.	6.6 ± 0.06 ^{abcd}	1.74 ± 0.01 ^{abcde}	1.08 ± 0.02 ^{ino}	35.43 ± 0.39 ^{lm}	27.75 ± 0.13 ^{fg}	8.82 ± 0.6 ^{b}	9.35 ± 0.01 ^{fg}	44.29 ± 0.33 ^{bh}	32.54 ± 0.21 ^{p}
Sonchus oleraceus L.	20.23 ± 0.54 ^{m}	2.19 ± 0.01 ^{cdef}	2.45 ± 0.05 ^{lmo}	28.66 ± 0.18 ^{cd}	14.35 ± 0.12 ^{no}	6.8 ± 0.07 ^{ghk}	9.51 ± 0.17 ^{fg}	57.6 ± 0.55 ^{ej}	24.85 ± 1.41 ^{def}
Taraxacum F.H.Wigg. sect. *taraxacum*	10.97 ± 0.22 ^{knop}	2.22 ± 0.01 ^{cdef}	1.33 ± 0.03 ^{hin}	33.7 ± 0.09 ^{delm}	23.64 ± 0.39 ^{ef}	7.84 ± 0.33 ^{bcg}	10.59 ± 0.17 ^{cl}	52.45 ± 0.15 ^{f}	20.85 ± 0.86 ^{ahi}
Umbilicus rupestris (Salisb.) Dandy	15.62 ± 0.01 ^{h}	1.75 ± 0 ^{abcde}	2.23 ± 0.01 ^{ino}	34.86 ± 0.7 ^{elm}	12.63 ± 0.16 ^{no}	10.39 ± 0.08 ^{i}	10.47 ± 0.12 ^{cl}	60.71 ± 0.32 ^{cj}	19.56 ± 0.25 ^{abh}
Urtica dioica L.	13.19 ± 0.47 ^{hijk}	2.84 ± 0.25 ^{f}	1.95 ± 0.17 ^{mno}	33.66 ± 0.15 ^{delm}	30.44 ± 1.33 ^{bcg}	8.1 ± 0.12 ^{bcg}	11.44 ± 0.06 ^{bk}	35.45 ± 0.14 ^{an}	25.33 ± 0.02 ^{def}
